# Attenuation of Compulsive-Like Behavior Through Positive Allosteric Modulation of α4β2 Nicotinic Acetylcholine Receptors in Non-Induced Compulsive-Like Mice

**DOI:** 10.3389/fnbeh.2016.00244

**Published:** 2017-01-05

**Authors:** Swarup Mitra, Mckenzie Mucha, Shailesh N. Khatri, Richard Glenon, Marvin K. Schulte, Abel Bult-Ito

**Affiliations:** ^1^Department of Chemistry and Biochemistry, University of Alaska FairbanksFairbanks, AK, USA; ^2^IDeA Network of Biomedical Research Excellence (INBRE), University of Alaska FairbanksFairbanks, AK, USA; ^3^Department of Pharmaceutical Sciences, Philadelphia College of Pharmacy, University of the SciencesPhiladelphia, PA, USA; ^4^Department of Medicinal Chemistry, School of Pharmacy, Virginia Commonwealth UniversityRichmond, VA, USA; ^5^Department of Biology and Wildlife, University of Alaska FairbanksFairbanks, AK, USA

**Keywords:** desformylflustrabromine (dFBr), α4β2 nicotinic receptors, positive allosteric modulator, obsessive compulsive disorder, non-induced compulsive-like mice

## Abstract

Nicotinic α4β2 receptors are the most abundant subtypes of nicotinic acetylcholine receptors (nAChRs) expressed in brain regions implicated in obsessive compulsive disorder (OCD). These receptors are known to modify normal and addictive behaviors by modulating neuronal excitability. Desformylflustrabromine (dFBr) is a novel, positive allosteric modulator (PAM) of high acetylcholine sensitivity (HS) and low acetylcholine sensitivity (LS) α4β2 nAChRs. The present study tested the hypothesis that positive allosteric modulation of α4β2 receptors by dFBr will attenuate compulsive-like behavior in a non-induced compulsive-like mouse model. Male mice (*Mus musculus*) selected for compulsive-like nesting behavior (NB; 48 animals; 12 per group) received acute (once) and chronic (every day for 32 days) subcutaneous injection of dFBr at 2, 4 and 6 mg/kg doses. Saline was used as a control (0 mg/kg). Compulsive-like NB was assessed after 1, 2, 3, 4, 5 and 24 h, while compulsive-like marble burying (MB) and anxiety-like open field (OF) behaviors were performed 2 h after dFBr administration. In the acute administration protocol, dFBr dose dependently attenuated NB and MB. Rapid effects (1–2 h after drug administration) of dFBr on MB and NB were observed for the chronic administration which was in congruence with the acute study. Chronic administration also revealed sustained suppression of NB by dFBr following 5 weeks of treatment. In both the acute and chronic regimen dFBr did not modulate OF behaviors. This research demonstrates the novel role of positive allosteric modulation of α4β2 nicotinic receptors by dFBr as a translational potential for OCD.

## Introduction

Obsessive-compulsive disorder (OCD) is the fourth most common mental disorder (Pittenger et al., 2005). It has a lifetime prevalence of 2.3% and a 12-month prevalence of 1.2% (Ruscio et al., 2010). Patients suffering from OCD suffer from persistent obsessive thoughts causing distress, and perform compulsive repetitive behaviors to alleviate uncomfortable feelings resulting from obsessions (Diniz et al., 2012). OCD can have disabling effects throughout the patient’s lifespan in both males and females (Attiullah et al., 2000).

Obsessions can be thematic, such as fear of contamination, pathological doubt, or need for symmetry/order, or somatic obsessions, like aggression. Repetitive compulsive behaviors involve, washing, seeking, counting, sorting, hoarding and searching (Doron and Moulding, 2009; Goit and Ghimire, 2014; Pauls et al., 2014). Although recently declassified as an anxiety disorder (American Psychiatric Association, 2015), many clinicians conceptualize OCD as a spectrum of related disorders (OCRD) sharing common clinical features of anxiety/fear and worry (Stein and Lochner, 2006; Storch et al., 2008; Fornaro et al., 2009). OCRD encompasses a wide range of diseases which includes somatoform (e.g., Hypochondriasis), impulse control (e.g., Trichotillomania, pathological gambling) and tic disorders (e.g., Tourette’s syndrome; Fornaro et al., 2009). Selective serotonin reuptake inhibitors (SSRIs) and cognitive behavioral therapy or their combination are often used as first line treatments. However, a large group of patients remain resistant to treatment either partially or completely (Jenike, 2004; Pittenger et al., 2005).

The cholinergic system in the brain is comprised primarily of nicotinic acetylcholine receptors (nAChRs; Paterson and Nordberg, 2000; Kalamida et al., 2007) and muscarinic acetylcholine receptors (mAChRs; Scarr, 2012; Thiele, 2013), members of the cys-loop superfamily of ligand gated ion channels and G protein-coupled receptors, respectively. Dysregulation of both mAChRs and nAChRs have been strongly associated with several neurological disorders (Janowsky et al., 1972; Freedman et al., 1995; Warpman and Nordberg, 1995; Breese et al., 2000; Salamone and Zhou, 2000; Perry et al., 2001; Woodruff-Pak and Gould, 2002; Ray et al., 2005; Quik et al., 2007; Scarr, 2012).

The α4β2 nAChR is one of the most prevalent nicotinic subtypes expressed in the brain (McGranahan et al., 2011). The α4β2 subtype is expressed in abundance in the dopamine pathways in the midbrain that influence the drug-induced reward system, mood disorders, stress, movement generation and learning (Wise, 2009; Maskos, 2010). α4β2 nAChRs have also been identified in the striatum, thalamus and cortex (Quik et al., 2013), brain areas implicated in OCD (Pena-Garijo et al., 2010; Fitzgerald et al., 2011). In the striatum, α4β2 receptors have also been shown to modulate GABA and dopamine release (McClure-Begley et al., 2009; Perez et al., 2012). In particular, a subtype of the α4β2 receptor with high sensitivity to acetylcholine (HS α4β2) appears to be involved in striatal dopamine release (Anderson et al., 2009). These studies support a modulatory role of α4β2 receptors in neurotransmitter release in circuits affected in OCD.

Positive allosteric modulators (PAMs) enhance agonist responses via increased agonist potency and/or efficacy. Desformylflustrabromine (dFBr) is a novel PAM capable of potentiating acetylcholine-induced whole cell responses by 370% for the HS and 260% for the low sensitivity (LS) α4β2 receptors with an EC_50_ of 40 μM and 2.5 μM respectively (Weltzin and Schulte, 2010, 2015). It is currently the only selective PAM for α4β2 receptors capable of potentiating the HS form of the receptor involved in striatal dopamine release. As dFBr increases the efficacy of acetylcholine and does not directly activate receptors, it is postulated that its effect in the synapse would be to enhance acetylcholine mediated transmission. Application of dFBr, unlike application of exogenous agonists, would thus retain the control of synaptic activation via presynaptic release of acetylcholine, albeit with increased stimulation (Weltzin and Schulte, 2015). Only one *in vivo* study has been conducted to examine the effect of dFBr potentiation of α4β2 nAChR in an *in vivo* behavioral model. In this study dFBr was shown to attenuate nicotine self-administration in rats (Liu, 2013). The use of HS α4β2 receptors PAMs for the treatment of OCD has not been previously proposed or tested in any animal model. The aim of the current study was to evaluate our hypothesis that acute and chronic administration of dFBr, a novel PAM specific for α4β2 nAChRs and active at the HS α4β2 subtype, will attenuate compulsive-like and anxiety-like behaviors in our non-induced compulsive-like mouse model.

There are few animal models that exhibit consistent and spontaneous differences in compulsive-like behaviors. We have previously shown that our mice exhibit face and predictive validity as a spontaneous non-induced model for OCD-like behaviors (Greene-Schloesser et al., 2011). The current model was achieved by bidirectionally selecting house mice, Mus musculus, for nest-building behavior for 56 generations (Lynch, 1980; Bult and Lynch, 2000). The stock population for the original selection experiment (Lynch, 1980) was a cross among eight inbred strains, i.e., A, AKR, BLB/c, C3H/2, C57BL, DBA/2, Is/Bi and RIII, to yield the HS/Ibg outbred strain (McClearn Ge and Meredith, 1970; Lynch, 1980). Bidirectional selection resulted in three levels of nesting behavior (NB). All BIG mice exhibit consistent excessive NB engaging in rapid and repetitive pulling of cotton through the cage top metal bars amounting to 6–7 g of cotton on an average in 24 h when compared to normal NB (no significant hyperactivity and repetitiveness when introduced to cotton averaging around 0.50–0.70 g in 24 h) by the Control strain (non-compulsive) and very little NB (most of them do not indulge in nesting) by the SMALL strain (non-compulsive). The Control mice therefore serve as a selection control with intermediate levels between compulsive-like BIG and non-compulsive SMALL strains (Bult and Lynch, 2000). NB is homologous to hoarding in humans with OCD (Warneke, 1993), which is considered to be a measure of compulsive-like phenotype in mice (Greene-Schloesser et al., 2011; Wolmarans De et al., 2016). The BIG mice also uniformly display repetitive marble burying (MB) behavior burying on an average 19–20 marbles. Both these behaviors are significantly attenuated by SSRIs (e.g., fluoxetine) used to treat OCD but not with normal antidepressants (e.g., desipramine; Greene-Schloesser et al., 2011) substantiating the face and predictive validity of the NB and MB phenotype of the BIG mice for investigating compulsive disorders. Hence in the current context of investigation compulsive-like BIG mice have been considered.

## Materials and Methods

### Animals

Compulsive-like BIG male mice, *Mus musculus*, were raised on wood shavings in polypropylene cages (27 cm × 17 cm × 12 cm) under controlled temperature (22 ± 1°C) and light (12:12 light-dark cycle) with free access to food (Purina Mills, Lab Diet Mouse Diet #5015, St. Louis, MO, USA) and water. Animals were 60 days of age at the start of the experiment. The University of Alaska Fairbanks Institutional Animal Care and Use Committee approved the animal care and experimental procedures (protocol # 675023).

### Drug Administration

Deformylflustrabromine hydrochloride (dFBr; Abcam Biochemicals) was dissolved in physiological saline (pH = 6.7) to yield final doses of 2 mg/kg (0.27 mg/mL), 4 mg/kg (0.53 mg/mL) and 6 mg/kg (0.80 mg/mL). Saline was used as a vehicle control (0 mg/kg). A mouse of 40 g received an injection volume of 0.3 mL. Injection volumes were proportionally adjusted according to the body weight of individual animals. All behaviors were performed in the light phase of the light:dark cycle. All data were recorded by an individual blinded to the study.

#### Acute Study

**Figure 1:**
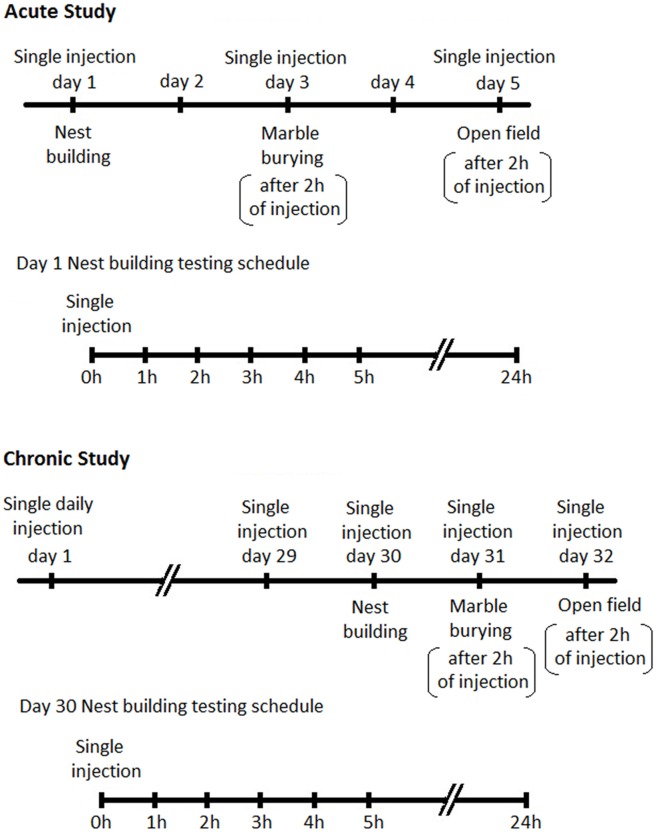
**Schedules for behavioral assessments following Desformylflustrabromine (dFBr) administration.** *Top panel-Acute Study*: mice in all experimental groups (0, 2, 4 and 6 mg/kg) received subcutaneous administration of vehicle or dFBr on days 1, 3 and 5. On day 1, immediately after injections all mice were subjected to nest-building and data were collected 1, 2, 3, 4, 5 and 24 h after injection (nest building testing schedule). On day 3 and 5 all mice were subjected to marble burying (MB) and open field (OF) behaviors, respectively, 2 h after vehicle or dFBr injections. On days 2 and 4 mice were not given injections and were not tested. *Lower panel-Chronic study*: for the chronic study mice from all groups (0, 2, 4 and 6 mg/kg) received daily single subcutaneous injections of vehicle or dFBr for 32 days. On day 30, immediately after injection all mice were subjected to nest-building and data were collected after 1, 2, 3, 4, 5 and 24 h after injection (nest building testing schedule). On day 31 and 32 all mice were subjected to MB and OF behaviors, respectively, 2 h after vehicle or dFBr injections.

Male BIG mice were divided into four treatment groups comprising vehicle (sterile saline), 2 mg/kg, 4 mg/kg and 6 mg/kg. Animals in each group (*n* = 12 per group) were tested for nesting on day 1, MB on day 3 and open field (OF) on day 5. On the first day of testing animals randomly received dFBr or vehicle subcutaneously and in subsequent tests received the same dose. Days 2 and 4 were employed to avoid any residual effects of dFBr from previous administration. For nesting, data were collected after 1, 2, 3, 4, 5 and 24 h due to the progressive nature of the NB (The BIG mice typically get excited and indulge in excessive and repetitive NB when introduced to cotton for the first 3–4 h in the light cycle. This excessive and repetitive nesting activity resumes again in the dark cycle). MB and OF behavior was performed 2 h after dFBr administration (Figure 1).

#### Chronic Study

Since the foundation of our animal model was established through effective reversal of compulsive-like NB and MB behaviors by chronic fluoxetine treatment (Greene-Schloesser et al., 2011) we also conducted a chronic regimen to establish the sustained and long term effects of dFBr on NB and MB. Animals belonging to 0, 2, 4 and 6 mg/kg dose group (*n* = 12 per group) received single subcutaneous injection of dFBr or saline daily for 32 days. NB, MB and OF behaviors were assessed in the final week (weeks 5) after dFBr administration (NB after 1, 2, 3 4, 5 and 24 h and MB after 2 h of drug injection). NB was performed on day 30, MB on day 31 and OF on day 32 (Figure 1).

The dosages and route of administration was determined based on a prior *in vivo* study of dFBr on rats (Liu, 2013). Studies on rats have shown that dFBr penetrates the blood-brain barrier and reaches the brain amounting to around 36% in the cerebrospinal fluid after 90 min of subcutaneous administration (Liu, 2013).

### Assessment of Compulsive-Like Behaviors

#### Nest-Building Behavior

Nest-building behavior (NB) was performed to assess compulsive-like behavior in the mice (Greene-Schloesser et al., 2011). For both the acute and chronic study, compulsive-like male mice were singly housed and provided with a pre-weighed roll of cotton (Mountain Mist cotton batting, Troy, Inc., Chicago, IL, USA) in the cage-top food hopper immediately following subcutaneous injection of dFBr. The cotton roll was weighed after 1, 2, 3, 4, 5 and 24 h. NB was quantified by the grams of cotton used during each testing period (Bult and Lynch, 1996, 1997, 2000; Greene-Schloesser et al., 2011).

#### Marble Burying Behavior

The MB test is an effective test for determining compulsive-like behavior in mice (Takeuchi et al., 2002; Thomas et al., 2009; Angoa-Pérez et al., 2013). Mice generally do not interact with the marbles and therefore the MB test measures only digging behavior (personal observations). Two hours after dFBr administration, compulsive-like male mice were individually introduced to a polypropylene cage (37 cm × 21 cm × 14 cm) containing 20 glass marbles (10 mm in diameter) evenly spaced on 5 cm deep bedding comprised of wood shavings without access to food or water for 20 min (Greene-Schloesser et al., 2011). Testing was carried out in the testing room separate from the housing room. The total number of marbles buried at least 2/3 in the 20-min period was quantified as compulsive-like digging behavior. After the 20-min test, the animals were returned to their home cages.

### Assessment of Locomotory and Anxiety-Like Behavior

#### Open Field Test

Anxiety-like behaviors were determined in the OF test (Simon et al., 1994; Prut and Belzung, 2003). Compulsive-like male mice were individually introduced into an OF (40 cm × 40 cm × 35 cm) with a central zone (20 cm × 20 cm). The apparatus was placed underneath an overhead light illuminating the entire OF (Greene-Schloesser et al., 2011). The animals were placed in the center of the OF and their behavior was video taped for 3 min and analyzed with the aid of ANYMaze^TM^ video tracking software (Stoelting Co., Wood Dale, IL, USA). The time spent in the center (anxiety-like measure) and total distance traveled (locomotion) in the entire OF were measured. The OF was cleaned before each test. Prior experiments (Greene-Schloesser et al., 2011) with the BIG mice in OF indicate that a 3 min duration provides consistent outcomes for assessment of locomotory and anxiety-like behaviors and therefore considered for the current experiment.

### Statistical Analysis

Statistical analysis was performed in Graphpad Prism (GraphPad Software, Inc.) and Statistical Analysis System Software (SAS Version 9.4, Cary, NC, USA). NB (grams of cotton), MB (number of marbles at least 2/3 buried) and OF measures (time in center and total distance traveled) were expressed as the mean ± standard error of the mean (SEM). The NB data were shown in figures as grams of cotton used, whereas the statistical analysis was conducted on the square-root transformed nesting scores in order to normalize the data (Bult and Lynch, 2000). Nesting scores at different time points, MB and OF results were analyzed by one-way analysis of variance (ANOVA) whereas, overall drug and drug by time interaction effect between 0 h and 5 h was done by two-way repeated ANOVA. Pairwise comparisons for significant differences between doses were tested by the *post hoc* Bonferroni multiple comparison test. A probability level of *p* < 0.05 was used as an index of statistical significance in all cases.

## Results

### dFBr Attenuates Compulsive-Like NB

#### Significant Suppression of Compulsive-Like NB During the First 5 h of Acute dFBr Administration (Figure 2A)

There was an overall significant drug (*F*_(3,220)_ = 38.60, *p* < 0.0001) effect during the first 5 h, a significant time (*F*_(4,220)_ = 44.71, *p* < 0.0001) and drug by time interaction effect (*F*_(12,220)_ = 7.08, *p* < 0.0001) in the compulsive-like NB.

**Figure 2:**
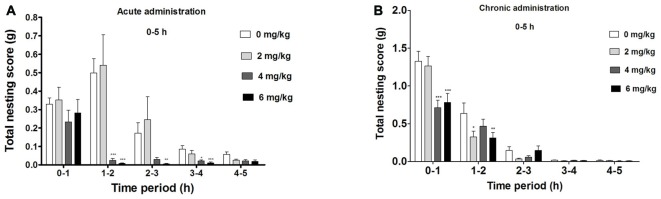
**Dose-dependent effect of dFBr on compulsive-like NB behavior in compulsive-like BIG mice (*n* = 12 in each group) from 1–5 h of** **(A)** acute and **(B)** chronic dFBr administration. Data are expressed as the mean ± standard error of the mean (SEM) for the amount of cotton used in grams. Statistical significance is considered as *p < 0.05, **p < 0.01 ***p < 0.001. All comparisons are with respect to control (saline).

Following a 1 h of dFBr administration, there was no significant attenuation of nesting (*F*_(3,44)_ = 1.276, not significant (NS)). Between 1 and 2 h, dFBr administration resulted in dose-dependent and significant reductions in nesting scores (*F*_(3,44)_ = 26.42, *p* < 0.0001). *Post hoc* assessment revealed that 4 mg/kg (*t*_22_ = 6.210, *p* < 0.001) and 6 mg/kg (*t*_22_ = 6.638, *p* < 0.001) doses of dFBr significantly attenuated NB as compared to the control (saline). This effect was sustained only by 6 mg/kg dose (*t*_22_ = 3.727, *p* < 0.01) between 2 and 3 h (*F*_(3,44)_ = 7.906, *p* < 0.0005) and both 4 and 6 mg/kg (*t*_22_ = 3.305, *p* < 0.05 and *t*_22_ = 4.585, *p* < 0.001 respectively) between 3 and 4 h (*F*_(3,44)_ = 8.094, *p* < 0.0005). Between 4 and 5 h after dFBr administration, nesting scores were not significantly different (*F*_(3,44)_ = 2.375, NS).

#### dFBr has an Overall Effect on NB Between 0 h and 24 h in the Acute Administration (Figure 3A)

Twenty four hours (time 0 through 24 h) after dFBr administration, overall nesting scores were dose-dependently and significantly reduced (*F*_(3,44)_ = 7.645, *p* < 0.001) with the 2 mg/kg (*t*_22_ = 6.213, *p* < 0.001), 4 mg/kg (*t*_22_ = 9.774, *p* < 0.001) and 6 mg/kg (*t*_22_ = 10.50, *p* < 0.001) groups significantly below the control group.

**Figure 3:**
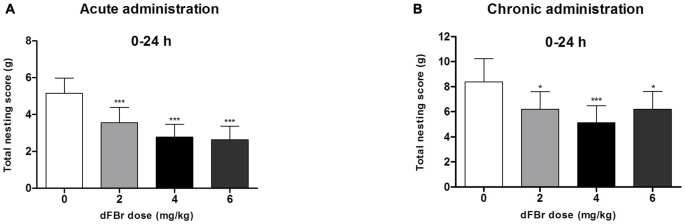
**Dose-dependent effects of dFBr on overall compulsive-like NB behavior in compulsive-like BIG mice (*n* = 12 in each group) 0–24 h after** **(A)** acute and **(B)** chronic dFBr administration. Data are expressed as the mean ± SEM for the amount of cotton used in grams. Statistical significance is considered as *p < 0.05 and ***p < 0.001. All comparisons are with respect to control (saline).

#### dFBr has an Overall Effect on NB During the First 5 h in the Chronic Administration (Figure 2B)

Significant drug (*F*_(3,220)_ = 4.87, *p* < 0.01) time (*F*_(4,220)_ = 177.12, *p* < 0.0001) and drug by time interaction effect (*F*_(12,220)_ = 2.29, *p* < 0.01) was observed in the first 5 h of the chronic administration.

Between 0 h and 1 h there was an overall suppression of NB (*F*_(3,44)_ = 6.52, *p* < 0.01) with 4 mg/kg and 6 mg/kg being the most effective doses (*t*_22_ = 6.097, *p* < 0.001 and *t*_22_ = 5.394, *p* < 0.001 respectively). For 1–2 h the NB declined (*F*_(3,44)_ = 4.86, *p* < 0.01) significantly with 2 and 6 mg/kg showing the main attenuating effects (*t*_22_ = 3.086, *p* < 0.05 and *t*_22_ = 3.210, *p* < 0.01 respectively). No significant effect was observed for NB between 2–3 (*F*_(3,44)_ = 1.54, NS), 3–4 (*F*_(3,44)_ = 1.01, NS) and 4–5 (*F*_(3,44)_ = 6.52, NS) h.

#### dFBr has an Overall Effect on NB Between 0 h and 24 h in the Chronic Administration (Figure 3B)

Twenty four hours (time 0 through 24 h) after dFBr administration, overall nesting scores were dose-dependently and significantly reduced (*F*_(3,44)_ = 8.85, *p* < 0.0001) with the 2 mg/kg (*t*_22_ = 4.574, *p* < 0.05), 4 mg/kg (*t*_22_ = 7.149, *p* < 0.001) and 6 mg/kg (*t*_22_ = 4.555, *p* < 0.05) groups significantly below the control group.

### dFBr Attenuates Compulsive-Like MB Behavior (Figure 4)

#### Acute Administration (Figure 4A)

MB behavior were significantly reduced (*F*_(3,44)_ = 64.62, *p* < 0.0001) 2 h after dFBr administration. The 2 mg/kg, 4 mg/kg and 6 mg/kg doses decreased MB dose-dependently compared to the control (*t*_22_ = 3.428, *p* < 0.01; *t*_22_ = 12.85, *p* < 0.001; *t*_22_ = 7.667, *p* < 0.001, respectively). The 4 mg/kg and 6 mg/kg doses also attentuated MB behavior more than the 2 mg/kg dose (*t*_22_ = 9.426, *p* < 0.001 and *t*_22_ = 5.332, *p* < 0.001, respectively).

**Figure 4:**
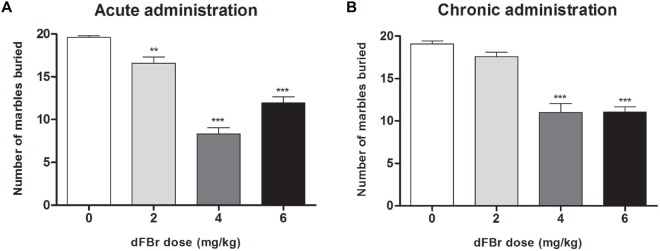
**Dose-dependent effect of dFBr on compulsive-like MB behavior in compulsive-like BIG mice (*n* = 12 in each group) 2 h after** **(A)** acute and **(B)** chronic dFBr administration. Data are expressed as the mean ± SEM for the number of marbles that are 2/3 buried. Statistical significance is considered as **p < 0.01 and ***p < 0.001. All comparisons are with respect to control (saline).

#### Chronic Administration (Figure 4B)

dFBr suppressed MB behavior significantly (*F*_(3,44)_ = 40.03, *p* < 0.0001) in the fifth week of administration. The most effective doses were 4 mg/kg and 6 mg/kg which showed the maximum suppression of MB when compared to control (*t*_22_ = 8.643, *p* < 0.001; *t*_22_ = 8.554, *p* < 0.001, respectively). The 4 and 6 mg/kg doses were also significantly lower than the 2 mg/kg dose (*t*_22_ = 7.039, *p* < 0.001; *t*_22_ = 6.950, *p* < 0.001, respectively).

### dFBr has no Effect on Anxiety-Like OF Behavior (Figure 5)

#### Acute Administration

**Figure 5:**
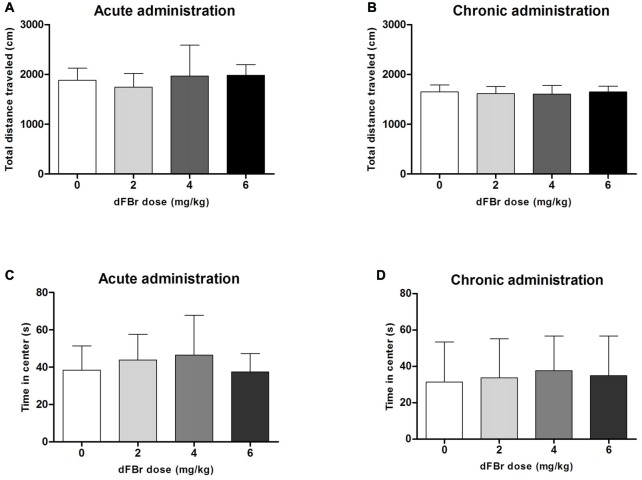
**Effect of dFBr on OF locomotory activity in** **(A)** acute administration and **(B)** chronic administration. Anxiety-like time in center in OF in **(C)** acute administration and **(D)** chronic administration in compulsive-like BIG mice (*n* = 12 in each group). Data are expressed as the mean ± SEM for the total distance traveled in the OF. No statistical significance was found.

The total distance traveled which is used to quantify locomotor activity was not different among the treatment groups (*F*_(3,44)_ = 1.213, NS; Figure 5A). No significant differences were also observed among the treatment groups for the time spent in center of the OF (*F*_(3,44)_ = 0.9849, NS; Figure 5C).

#### Chronic Administration

For the chronic regimen the total distance (*F*_(3,44)_ = 0.30, NS) and time in center (*F*_(3,44)_ = 0.18, NS) did not differ among treatment groups (Figures 5B,D).

## Discussion

Evidence exists for cholinergic involvement in OCD (Lucey et al., 1993; Yankelevitch-Yahav and Joel, 2013). Some studies have indicated exacerbation of OCD symptoms induced by nicotine (Abramovitch et al., 2015). In contrast to the higher rates of smoking in patients with psychiatric disorders, such as schizophrenia, bipolar disorder and ADHD, OCD patients report less smoking behavior (Bejerot and Humble, 1999; Bejerot et al., 2000; McCabe et al., 2004; Abramovitch et al., 2014). It has been suggested that nicotinic activation of an already hyperactivated fronto-striatal circuit worsens OCD symptoms (Abramovitch et al., 2015). However, other studies have shown that nicotine augmentation improves clinical symptoms in patients with OCD (Carlsson, 2001; Pasquini et al., 2005). Glutamatergic hyperactivity associated with OCD may also be due to mediation of glutamate release by nicotinic receptor activation. (Araki et al., 2002; Mansvelder et al., 2002; Pasquini et al., 2005). Studies investigating cholinergic involvement in glutamatergic hyperactivation suggest that nicotine promotes glutamatergic transmission and stabilizes hyperactivity of the neural circuit that originates in the orbitofrontal cortex and projects to the cingulate gyrus, the striatum and the thalamus (Pasquini et al., 2005). PET and fMRI studies in OCD subjects have shown elevated cerebral blood flow, metabolism and activation (indicators of hyperactivity) in the orbitofrontal cortex and amygdala in OCD (Busatto et al., 2000; Carlsson, 2000; Menzies et al., 2008). These regions receive substantial cholinergic innervations (Mesulam et al., 1986; Carlsson, 2000). Based on these prior studies, we investigated the modulatory role of α4β2 nAChRs in compulsive-like and anxiety-like behaviors in the compulsive-like mice model.

Administration of the novel α4β2 PAM, dFBr produced a reduction in compulsive-like NB and MB, but did not alter anxiety-like and locomotor activity in the OF for the acute study. A very similar response to chronic dFBr was observed where the treatment groups showed rapid suppression of NB (1 h and 2 h) and MB (2 h) after dFBr administration. OF behaviors however remained unaffected by the chronic treatment. These results indicate an apparent selectivity of dFBr for compulsive-like behaviors corroborating the hypothesis that potentiation of α4β2 nAChRs could be an alternative approach for suppressing compulsive-like phenotype thereby posing significant translational potential.

In the acute administration, 4 mg/kg and 6 mg/kg dFBr doses had the largest attenuating effects on NB 2 h after injection, while for the chronic administration the suppression effects on NB was visible after the first hour and endured in the second hour with 6 mg/kg showing a more consistent effect. Interestingly, an earlier effect of dFBr (1 h after administration) on NB was observed for the chronic study indicating potential sensitization to dFBr due to repeated treatment. The attenuating effects gradually decreased during the next 4 h for both the treatment, showing that dFBr had a rapid effect. This result is consistent with the finding that peak levels of dFBr in the cerebrospinal fluid occur 90 min after administration in rats (Liu, 2013). The 2 mg/kg dFBr dose had no immediate attenuating effect on NB. A long term effect of this dose was however seen in both acute (after 24 h) and chronic (week 5) administration indicating that this dose was effective over a longer time period.

The effects of dFBr, 2 h after injection on MB behavior were generally similar to the effects on NB. However, at the 2 h time point in the acute treatment 2 mg/kg moderately and significantly reduced MB behavior. This effect was not significant in the chronic regimen. No significant effect was observed on NB at the same dose and time point in the acute study but had an effect in the chronic study. These different effects of dFBr treatment may indicate subtle differences in the brain mechanisms that control NB and MB behavior. Clinical studies have shown that some OCD patients with specific types of symptoms do not respond to first line therapies in a similar way (McKay et al., 2004). The doses that act to attenuate obsessions and compulsions in general OCD patients typically fail to produce results in treatment resistant ones (Albert et al., 2013). Moreover, recommended doses for first line treatments might vary depending on the severity of the disorder, co-morbid symptoms like anxiety and potential side effects (Hanna et al., 2011; Albert et al., 2013). Though, a common agreement on OCD subtypes is lacking, therapeutic response and results for each OCD subtype are different (Alonso et al., 2001). For example, fluoxetine, a common OCD drug has greater efficacy in washers and obsessive thoughts when compared to checkers (Farnam et al., 2008). Therefore, the variation in dose response to dFBr of compulsive-like MB and NB behavior adds additional heterogeneity to the BIG mouse for assessing drug effects on various compulsive-like phenotypes.

Acute and chronic dFBr regimen failed to modulate anxiety-like (time spent in center) and locomotor (total distance traveled) behaviors in the OF test. Previous studies using the BIG mice have shown a similar effect of SSRIs like fluoxetine, which failed to reduce overall wheel-running locomotion in the compulsive-like BIG mice but significantly attenuated NB and MB behavior (Greene-Schloesser et al., 2011). Separate brain regions and signaling pathways influencing compulsive-like and anxiety-like symptoms are most likely the explanation for the observed lack of a dFBr effect in the OF test. Anxiety is attributed primarily to the amygdala and ventral hippocampus (McHugh et al., 2004), whereas compulsions and obsessions have been linked to dorsolateral prefrontal cortex (Hirosawa et al., 2013), anterior cingulate cortex (Fitzgerald et al., 2005), orbitofrontal cortex (Beucke et al., 2013) and dysregulation of the corticostriatal-thalamo-cortical circuitry (CSTC; Ting and Feng, 2011). These regions receive projections from the amygdala and hippocampus (McDonald, 1991; Eblen and Graybiel, 1995; Welch et al., 2007; Toyoda et al., 2011; Chen and Etkin, 2013) explaining the co-existence of anxiety along with OCD, which appears to be specific to anxiety related to compulsive-like behaviors rather than more generalized anxiety.

Removal or inhibition by antagonists of α4β2 nAChRs abolishes the anxiolytic effects of nicotine, while stimulating these nAChRs receptors with an agonist decreases anxiety-like behavior. In contrast, anxiogenic effects of nicotine withdrawal are enhanced by stimulation of α7 nAChRs and decreased by inhibition of these nAChRs receptors (Kutlu and Gould, 2015). Allosteric modulation of α4β2 nAChRs by dFBr did not affect anxiety-like behavior in the OF test in the BIG mice, suggesting that these nAChRs receptors may not be involved in the control of anxiety in nicotine-naïve mice. A partial agonist of α4β2 nAChRs (ABT-089) caused anxiogenic effects in nicotine-naïve mice (Yohn et al., 2014). Whether this result contradicts our findings or could be due to low affinity of ABT-089 for α7 nAChRs remains to be determined.

In summary, both acute and chronic dFBr was effective in reversing compulsive-like NB and MB, without exerting any influence on anxiety-like and locomotory behaviors. This indicates the therapeutic potential of modulation of α4β2 nAChRs by dFBr for compulsive phenotypes. Due to the rapid rate of onset (a few hours) of the attenuating effects of dFBr on compulsive-like behaviors, this class of specific nicotinic subtype modulators might also provide more immediate suppression effects thereby provide a bridging option to other first line therapies (e.g., SSRIs) that display longer time courses for onset of effectiveness. dFBr maintained its attenuating effects on NB and MB during chronic treatment, and may therefore also represent a novel first line treatment. However, the cellular mechanisms leading to such acute and chronic suppression of compulsive-like behavior and the role of upstream and downstream targets that ultimately modulate phenotypic expression of the behaviors remains to be elucidated. It also remains to be determined if this effect of dFBr is consistent across all rodent models of compulsive-like phenotype. The current study thereby provides a strong impetus for further exploration of these factors in otherwise sparsely explored area of the role of nAChRs in OCD.

## Author Contributions

SM, MM and SNK conducted all experiments and performed data analysis. SM and SNK lead manuscript writing efforts. AB-I, RG and MKS made significant contributions to research design, data interpretations and manuscript preparation.

## Funding

Research reported in this publication was supported by an Institutional Development Award (IDeA) from the National Institute of General Medical Sciences of the National Institutes of Health under grant number P20GM103395 to SM and AB-I. This work was also supported by an Undergraduate Research and Scholarly Activity grant (#S15-24) to MM. The College of Natural Sciences and Mathematics also supported this work. These funding sources did not have a role in the study design, collection, analysis and interpretation of data and submission of this article for publication.

## Conflict of Interest Statement

The authors declare that the research was conducted in the absence of any commercial or financial relationships that could be construed as a potential conflict of interest.
